# SARS-CoV-2 Seroprevalence after Third Wave of Infections, South Africa

**DOI:** 10.3201/eid2805.220278

**Published:** 2022-05

**Authors:** Jackie Kleynhans, Stefano Tempia, Nicole Wolter, Anne von Gottberg, Jinal N. Bhiman, Amelia Buys, Jocelyn Moyes, Meredith L. McMorrow, Kathleen Kahn, F. Xavier Gómez-Olivé, Stephen Tollman, Neil A. Martinson, Floidy Wafawanaka, Limakatso Lebina, Jacques D. du Toit, Waasila Jassat, Mzimasi Neti, Marieke Brauer, Cheryl Cohen, for the PHIRST-C Group

**Affiliations:** National Institute for Communicable Diseases of the National Health Laboratory Service, Johannesburg, South Africa (J. Kleynhans, S. Tempia, N. Wolter, A. von Gottberg, J.N. Bhiman, A. Buys, J. Moyes, W. Jassat, M. Neti, C. Cohen);; University of the Witwatersrand, Johannesburg (J. Kleynhans, S. Tempia, N. Wolter, A. von Gottberg, J.N. Bhiman, J. Moyes, C. Cohen);; US Centers for Disease Control and Prevention, Atlanta, Georgia, USA (S. Tempia, M.L. McMorrow);; US Centers for Disease Control and Prevention, Pretoria, South Africa (M.L. McMorrow);; MRC/Wits Rural Public Health and Health Transitions Research Unit (Agincourt), University of the Witwatersrand, Johannesburg (K. Kahn, F.X. Gómez-Olivé, S. Tollman, F. Wafawanaka, J. du Toit);; Johns Hopkins University Center for Tuberculosis Research, Baltimore, Maryland, USA (N.A. Martinson);; Perinatal HIV Research Unit, University of the Witwatersrand, Johannesburg (N.A. Martinson, L. Lebina);; Africa Health Research Institute, Durban, South Africa (L. Lebina);; Ampath Pathology, Pretoria (M. Brauer)

**Keywords:** COVID-19, coronavirus disease, SARS-CoV-2, severe acute respiratory syndrome coronavirus 2, viruses, respiratory infections, zoonoses, vaccine-preventable diseases, seroprevalence, case-to-infection ratio, hospitalization-to-infection ratio, fatality-to-infection ratio

## Abstract

By November 2021, after the third wave of severe acute respiratory syndrome coronavirus 2 infections in South Africa, seroprevalence was 60% in a rural community and 70% in an urban community. High seroprevalence before the Omicron variant emerged may have contributed to reduced illness severity observed in the fourth wave.

South Africa has experienced 4 waves of severe acute respiratory syndrome coronavirus 2 (SARS-CoV-2) infections, the fourth dominated by the Omicron variant of concern (*1*). Data on the proportion of the population with serologic evidence of previous infection at the time of Omicron emergence are important to contextualize the observed rapid increases and subsequent quick decline in case numbers (*1*), as well as the lower severity compared with previous variants (*2*).

We previously described the seroprevalence of SARS-CoV-2 in the PHIRST-C (Prospective Household Study of SARSCoV-2, Influenza, and Respiratory Syncytial Virus Community Burden, Transmission Dynamics, and Viral Interaction) cohort in a rural and an urban community at 5 timepoints during July 2020–March 2021 (*3*). By using the same methods (Appendix), we report seroprevalence at 4 additional timepoints through November 27, 2021, spanning the third, Delta-dominated wave (Appendix Figure 1), ending the week Omicron was identified (*4*). We tested serum samples by using the Roche Elecsys Anti-SARS-CoV-2 assay (Roche Diagnostics, https://www.roche.com); we considered a cutoff index >1.0 an indication of prior infection. The immunoassay detects nucleocapsid (N) antibodies; thus, it does not detect postvaccination antibody responses. We obtained seroprevalence 95% credible intervals (CrIs) by using Bayesian inference with 10,000 posterior draws (*5*). We estimated the age- and sex-adjusted number of infections and age-adjusted diagnosed cases, hospitalizations, deaths, case-to-infection ratio (CIR), hospitalization-to-infection ratio (HIR), and in-hospital and excess death fatality-to-infection ratio (FIR), as described previously (*3*) (Appendix). Third-wave infections were defined as participants who had a paired blood draw (BD) from the fifth timepoint of the previous study (BD5) (collected March 22–April 11, 2021) and from the ninth timepoint of this study (BD9) (collected November 15–27, 2021) and who were seronegative at BD5 and seropositive at BD9 or seropositive at BD5 but had a >2-fold higher cutoff index in BD9 (because 38 possible reinfections occurred after BD5 [Appendix]). We obtained vaccination status through reviewing vaccine cards that participants kept at home. The study was approved by the University of the Witwatersrand Human Research Ethics Committee (reference no. 150808); the US Centers for Disease Control and Prevention relied on local clearance (IRB approval no. 6840).

Overall, pre–third wave (BD5) SARS-CoV-2 seroprevalence adjusted for assay sensitivity and specificity was 26% (95% CrI 22%–29%) in the rural and 41% (95% CrI 37%–45%) in the urban community. After the third wave (BD9), overall seroprevalence increased to 60% (95% CrI 56%–64%) in the rural community and 70% (95% CrI 66%–74%) in the urban community (Figure; Appendix Table 1). In both communities, the largest increase in seroprevalence was seen in children 13–18 years of age, who also had the highest seroprevalence of all ages after the third wave: 80% (95% CrI 70%–88%) in the rural community (a 49% increase) and 83% (95% CrI 73%–90%) in the urban community (a 19% increase).

**Figure:**
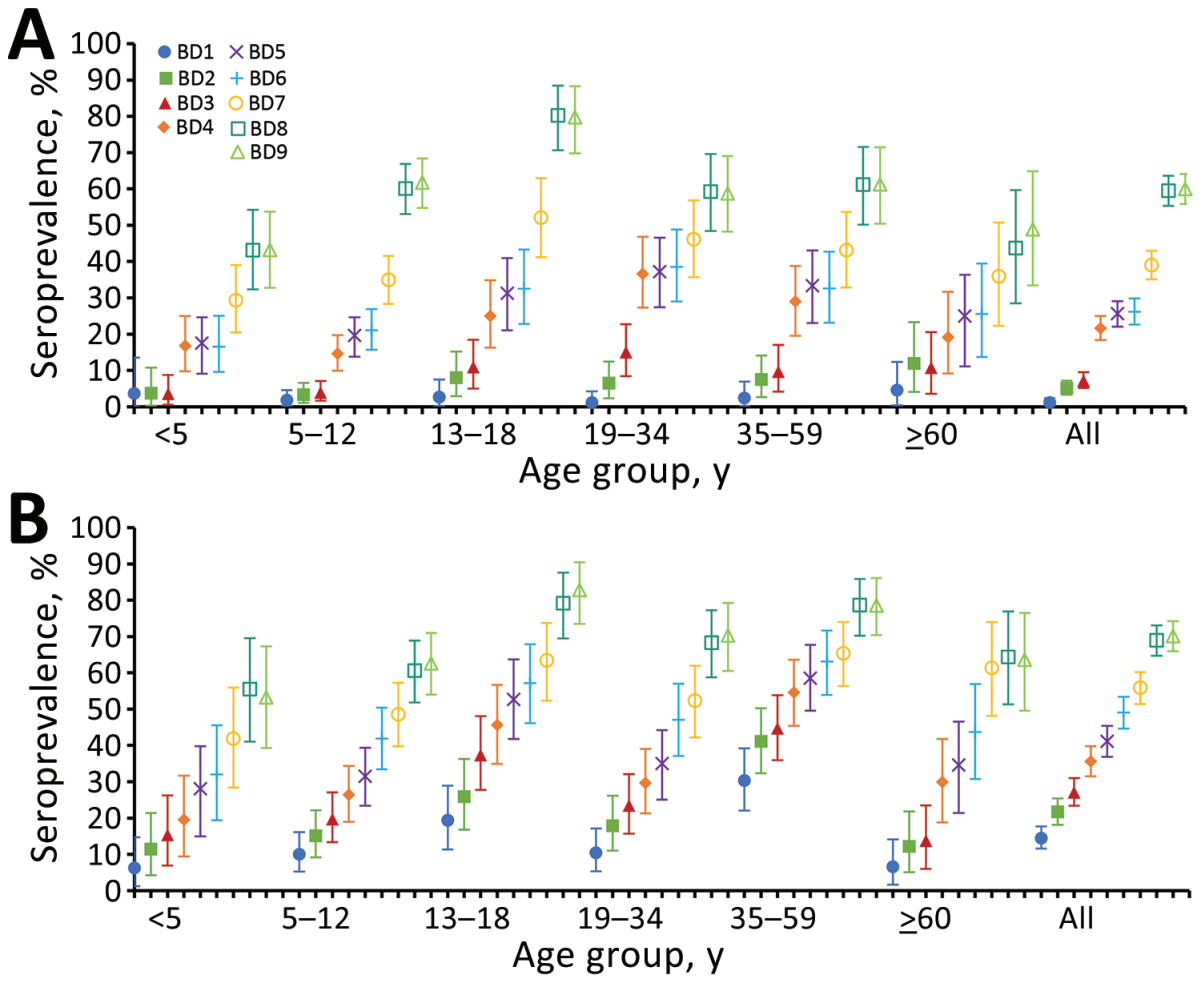
Severe acute respiratory syndrome coronavirus 2 seroprevalence at each blood collection, by age group, in a rural community (A) and urban community (B), South Africa, March 2020–November 2021. Baseline blood draw (BD1) collected July 20–September 17, 2020; second draw (BD2), September 21 – October 10, 2020; third draw (BD3), November 23–December 12, 2020; fourth draw (BD4), January 25–February 20, 2021; fifth draw (BD5), March 22–April 11, 2021; sixth draw (BD6), May 20–June 9, 2021; seventh draw (BD7), July 19–August 5, 2021; eighth draw (BD8), September 13–25, 2021; ninth draw (BD9), November 15–27, 2021. Error bars represent 95% credible intervals. Seroprevalence estimates adjusted for sensitivity and specificity of assay.

During the third wave of infections, the incidence at the rural site was 39% (95% CrI 24%–55%), resulting in a CIR of 3% (95% CI 2%–5%). HIR was 0.5% (95% CI 0.3%–0.7%) and in-hospital FIR was 0.1% (95% CI 0.1%–0.2%); excess deaths FIR was 0.5% (95% CI 0.4%–0.8%) (Figure; Appendix Figure 2).

In the urban community, the incidence during the third wave was 40% (95% CrI 26%–54%). CIR was a 5% (95% CI 4%–8%), and HIR was 2% (95% CI 2%–4%). In-hospital FIR was 0.4% (95% CI 0.3%–0.6%) and excess deaths FIR was 0.6% (95% CI 0.4%–0.9%) (Figure; Appendix Figure 2).

HIR and FIR were similar between wave 2 and 3 (Appendix Figure 3). SARS-CoV-2 vaccines became available in South Africa in February 2021, after the second wave. By the end of wave 3, only 8% (49/609) of participants were fully vaccinated (1 dose of Johnson & Johnson/Janssen or 2 doses of Pfizer-BioNTech) in the rural community and 19% (97/512) in the urban community (Appendix Table 2). Considering the overall low vaccination coverage in these communities during the study period, the similar HIR and FIR in wave 2 and 3 were likely driven by a combination of natural immunity and potentially a moderate effect attributable to vaccination.

Taken together, by the end of November 2021, just before the emergence of Omicron, the combined proportion of persons who had serologic evidence of previous infection (at any timepoint), were fully vaccinated, or both was 62% (389/631) at the rural community and 72% (411/568) at the urban community (Appendix Table 3).

After the third wave of infections in South Africa, we observed a >60% overall seroprevalence attributable to SARS-CoV-2 infection, ranging from 43% in rural community children <5 years of age to 83% in urban community children 13–18 years of age (Figure). CIR, HIR, and FIRs were similar between the second and third waves. Similar to our data, results from a study in Gauteng Province found seroprevalence of 56%–80% attributable to natural infection before the emergence of Omicron (*6*). The high seroprevalence before Omicron emergence may have contributed to reduced illness severity observed in the fourth wave (*2*). 

Appendix Additional information about SARS-CoV-2 seroprevalence in a rural and urban community household cohort after the third wave of infections, South Africa, April–November 2021.
