# Supplementary material for: SARS-CoV-2 Seroprevalence after Third Wave of Infections, South Africa
Source: Emerg Infect Dis. 2022 May;28(5):1055–8. doi: 10.3201/eid2805.220278 (PMC9045427; doi:10.3201/eid2805.220278)
Supplement: Appendix — Additional information about SARS-CoV-2 seroprevalence in a rural and urban community household cohort after the third wave of infections, South Africa, April–November 2021. [file 22-0278-Techapp-s1.pdf]

Article DOI: https://doi.org/10.3201/eid2805.220278

SARS-CoV-2 Seroprevalence after Third Wave of Infections, South Africa

Appendix

Supplementary Methods

Study Population

PHIRST-C (A Prospective Household study of SARS-CoV-2, Influenza, and Respiratory Syncytial virus community burden, Transmission dynamics and viral interaction in South Africa) is a continuation from PHIRST (A Prospective Household observational cohort study of Influenza, Respiratory Syncytial virus and other respiratory pathogens community burden and Transmission dynamics in South Africa) (*1*). PHIRST-C is conducted in two communities with established influenza-like illness and pneumonia surveillance sites.

South Africa consists of nine provinces, which are divided into 52 districts, which form the second level of administration. Districts are further divided into municipalities. The rural site is located in the Bushbuckridge Municipality, Ehlanzeni District, Mpumalanga Province and forms part of a health and sociodemographic surveillance site (HDSS) at the Medical Research Council (MRC)/University of Witwatersrand Rural Public Health and Health Transitions Research Unit, Agincourt. During PHIRST, two of the 29 villages within the HDSS were selected according to convenience (proximity and burden of other studies within the site) each year between 2016–2018. A random selection of ≈50 households with a household size of 3 or more were approached for enrolment. The urban site is located in the Jouberton Township, Matlosana Municipality, Dr Kenneth Kaunda District, North West Province. A list of 450 global positioning system (GPS) coordinates were generated using Google Earth. Study staff approached the nearest house within 30 m of the coordinate point for enrolment. Approximately 50 households were enrolled each year during 2016–2018. At both sites, households were eligible if they consisted of 3 or more household members (sharing at least four meals a week).

All household members were approached for inclusion in the study, and households were eligible for inclusion if >80% of members consented to be enrolled.

For PHIRST-C, all households who participated in PHIRST from the urban site, and households from the 2017 and 2018 cohort in the rural site were approached for enrolment. To supplement the sample size, additional households at each site were approached using the same methods as for PHIRST. Villages in the rural site were restricted to those four used in 2017 and 2018 of PHIRST. Informed consent was obtained from participating adults or a parent/guardian for children <18 years of age. In addition to parent/guardian consent, assent was also obtained from children aged 7–17 years.

We collected baseline data and blood (blood draw [BD] 1) at enrollment (July 20–September 17, 2020) and every 2 months thereafter: BD2, September 21–October 10; BD3, November 23–December 12, 2020; BD4, January 25–February 20, 2021; BD5, March 22–April 11, 2021; BD6, May 20 – June 9, 2021; BD7, July 19 – August 5, 2021; BD8, September 13 – 25, 2021; BD9, November 15 – 27, 2021.

The Bushbuckridge Municipality within the Ehlanzeni District is considered as predominantly rural, with main industries being agriculture and tourism (*2*). The Ehlanzeni District has a population of 1,828,738, with sixty percent of the district's population being >18 years of age. The City of Matlosana Municipality is considered to be 88.2% urban, and is located in the Dr Kenneth Kaunda District, which has a population of 797,715 (*3*). Sixty-five percent of the district population is aged >18 years.

Calculation of Case-to-infection Ratio (CIR), Hospitalization-to-infection Ratio (HIR), and Fatality-to-infection Ratio (FIR) by Wave of Infection

We calculated the age- and sex-adjusted total number of infections, age-adjusted diagnosed cases, hospitalizations, deaths, CIR (number of infections compared to diagnosed cases), HIR and in-hospital and excess death FIR as described in below equations. We defined the third wave as March 22, 2021 (week 12) to November 14, 2021 (week 45), starting with BD5, and ending the week before BD9 started. Due to differences between the sex ratios of our cohort and the district population, the infection estimates were also adjusted for sex. Data sources used in these calculations are described in the next section. Age- and sex- standardized estimates for the selected endpoints for each wave were obtained as follows:

$$Infections = \frac{\sum_i (s_i \times SAp_i)}{\sum_i (SAp_i)} \times 100{,}000$$

Where s_i is the seroprevalence in the cohort in the respective community for age and sex group i and SAp_i is the South African population for age and sex group i. Calculated for wave 3 as the number of individuals seronegative at BD5 and seropositive at BD9. Furthermore, we also included possible re-infections where the individual was already seropositive at BD5, but had a ≥2-fold higher cutoff index (COI) in BD9 compared to BD5. We therefore included infections and re-infections that occurred during the third wave. Estimates only included participants with a blood draw 5 and 9 pair, and adjusted for sensitivity and specificity of test (*4*).

The ≥2-fold increase in COI cutoff to define possible re-infections was not externally validated, but based on PCR-confirmed infections from twice-weekly nasal swab collections between March 22, 2021 (BD5) to July 19, 2021 (BD7) in the same cohort (C. Cohen et al., unpub. data, https://doi.org/10.1101/2021.07.20.21260855). Increases in COI from BD5 to BD7 ranged from 1 to 93 fold. Of the 30 individuals who had a rRT-PCR-confirmed infection between BD5 and BD7, 16 (53%, sensitivity) had a ≥2-fold increase from BD5 to BD7, and of the 312 individuals that did not have a rRT-PCR infection, 301 (96%, specificity) did not have a ≥2-fold increase. We were therefore more likely to have underestimated re-infections.

$$Diagnosed\ cases = \frac{\sum_i \left((c_i \div p_{i(d)}) \times p_{i(SA)} \right)}{\sum_i \left(p_{i(SA)} \right)} \times 100{,}000$$

Where c_i is the number of diagnosed cases (RT-PCR and antigen-based tests) from the respective district reported to the NMCSS during wave 3 (March 22, to November 14, 2021) in age group i, $p_{i(d)}$ is the district population for age group i and $p_{i(SA)}$ is the South African population for age group i.

$$Hospitalizations = \frac{\sum_i \left((h_i \div p_{i(d)}) \times p_{i(SA)} \right)}{\sum_i \left(p_{i(SA)} \right)} \times 100{,}000$$

Where h_i is the number of hospitalizations from the respective district reported to COVID-19 Sentinel Hospital Surveillance (DATCOV) during wave 3 (March 22, to November 14, 2021) (*5*) in age group i, $p_{i(d)}$ is the district population for age group i and $p_{i(SA)}$ is the South African population for age group i.

$$In - hospital\ deaths = \frac{\sum_i \left((d_i \div p_{i(d)}) \times p_{i(SA)} \right)}{\sum_i \left(p_{i(SA)} \right)} \times 100{,}000$$

Where d_i is the number of in-hospital deaths from the respective districts reported to DATCOV during wave 3 (March 22, to November 14, 2021) (*5*) in age group i, $p_{i(d)}$ is the district population for age group i and $p_{i(SA)}$ is the South African population for age group i.

$$Excess\ deaths = \frac{(ExD\ x\ 0.85)\ \times\ p_{(SA)}}{p_{(SA)}} \times 100{,}000$$

Where ExD is the rate of provincial excess deaths adjusted to the South African population reported by South African Medical Research Council during wave 3 (March 22, to November 14, 2021) (*6*), and $p_{(SA)}$ is the total South African population. According to estimates only 85% of excess deaths are attributable to COVID-19 (*6*).

For infections, hospitalizations, in-hospital (minimum) and excess (maximum) deaths, 95% CIs were calculated using the Clopper-Pearson method with a Poisson distribution.

$$Case - to - infection\ ratio\ (CIR) = \frac{Diagnosed\ cases}{Infections}\ x\ 100$$

$$Hospitalization - to - infection\ ratio\ (HIR) = \frac{Hospitalizations}{Infections}\ x\ 100$$

$$Fatality\ - to - infection\ ratio\ (FIR) = \frac{Deaths\ *}{Infections}\ x\ 100$$

Confidence intervals for infection ratios were calculated as ratios from the 95% confidence intervals of infection, hospitalization and death rates.

Data Sources

Population Denominators (*7*)

Population numbers for each district, by age, were obtained from the StatsSA 2021 mid-year population estimates.

Notifiable Medical Conditions Surveillance System (NMCSS) (*8*)

Reverse transcription PCR (RT-PCR) testing in South Africa to detect SARS-CoV-2 RNA started on 28 January 2020. Rapid SARS-CoV-2 antigen testing was implemented in

November 2020. All laboratories in the private and public sector performing SARS-CoV-2 tests automatically feed testing data from their lab information systems to the NMCSS, from where daily and weekly reporting on national, provincial and district SARS-CoV-2 infections are performed. No serology tests are used for this reporting. Results from antigen tests may be underestimated in the NMCSS as not all antigen test results are captured on lab information systems which feed into NMCSS. The number of cases reported to the NMCSS in the Ehlanzeni District (rural community) and Dr Kenneth Kaunda District (urban community) was used as an estimate of reported, diagnosed SARS-CoV-2 infections.

COVID-19 National Hospital Surveillance (DATCOV) (*5*)

DATCOV is a national hospital surveillance system to which all hospitals where COVID-19 admissions occurred in the public and private sector in South Africa report. The case definition includes any person admitted with a positive RT-PCR test result for SARS-CoV-2; and may include individuals for whom the main cause of hospitalization was not SARS-CoV-2. In-hospital outcome was available for all patients. The total number of hospitalizations and in-hospital deaths in each district reported to DATCOV between March 22, to November 14, 2021 was used to calculate in the HIR and minimum FIR, respectively.

South African Medical Research Council (SAMRC) Report on Weekly Deaths (*6*)

The Burden of Disease Unit at the SAMRC produces a weekly report on excess deaths in South Africa. Deaths in South Africa are registered on the Department of Home Affair's National Population Register and includes all citizens with a South African identification number. The number of excess deaths were defined as the number of all-cause deaths in that week minus the number of deaths expected for the week based on 2014–2019 trends. Excess death estimates are adjusted for incomplete reporting, and death estimates in provinces are age-standardized to the national population. Eighty-five percent of the reported excess deaths were used to calculate the maximum FIR as suggested by SAMRC.

Seroreversions

Seven individuals were seronegative at BD5, and with at least one seropositive result during BD6 and BD8, and who were seronegative again at BD9. The maximum COIs for these individuals were low, ranging from 1.09 to 34.5. This fits with an initial low antibody response and subsequent antibody titer waning. In addition, there were five individuals who were

seropositive at BD5, whose antibody titers waned below detection during BD6 to BD8, but who were seropositive again at BD9. Two of these individuals had a lower COI in BD9 than in BD5, and were not included as possible re-infections during wave 3.

Limitations

Our study is limited by a small sample size, reducing the power for accurate seroprevalence estimates in small age strata, and inclusion of only 2 geographic sites, including only households with ≥3 people, with high unemployment in selected households compared to the community unemployment rate (*9*) and therefore may not be representative of other districts and provinces in South Africa. Our definition of re-infections has not been validated elsewhere and is based on a small number of infections that occurred between BD5 and BD7, and did not consider different variants. Based on data from the same cohort, these reinfections occurred in only a small portion (3%) of the cohort (C. Cohen et al., unpub. data, https://doi.org/10.1101/2021.07.20.21260855) and would have had a negligible influence on the infection ratios. CIR, HIR, and FIR formed part of an ecologic analysis, which is inherently prone to biases. Transmission dynamics within our cohort may not be similar to the transmission dynamics within the district. Twenty percent (260/1,277) of persons did not have a BD 5+9 pair, and bias could have been introduced if the seroprevalence were different for those without a BD 5+9 blood pair. Since we did not have complete serologic results for draws between BD5 and BD9 for all individuals with a BD 5+9 pair, we did not consider individuals who may have seroreverted during wave 3 as infections or re-infections. This may have led to an underestimation of infections, and overestimation of the CIR, HIR and FIR.

References

1. Cohen C, McMorrow ML, Martinson NA, Kahn K, Treurnicht FK, Moyes J, et al.; PHIRST group. Cohort profile: a prospective household cohort study of influenza, respiratory syncytial virus and other respiratory pathogens community burden and transmission dynamics in South Africa, 2016–2018. Influenza Other Respir Viruses. 2021;15:789–803. PubMed https://doi.org/10.1111/irv.12881

2. Bushbuckridge Local Municipality. Bushbuckridge local municipality final integrated development plan, 2020/21. 2020 [cited 2021 Feb 16]. https://www.cogta.gov.za/cgta_2016/wp-content/uploads/2021/02/Bushbuckridge-Municipality.pdf

3. City of Marlosana. Matlosana spatial development framework. 2009 [cited 2021 Feb 16]. http://www.matlosana.gov.za/Documents/Policies/SPLUMA/SDF%20FRAMEWORK%202009.pdf

4. Larremore DB, Fosdick BK, Bubar KM, Zhang S, Kissler SM, Metcalf CJE, et al. Estimating SARS-CoV-2 seroprevalence and epidemiological parameters with uncertainty from serological surveys. eLife. 2021;10:e64206. PubMed https://doi.org/10.7554/eLife.64206

5. National Institute for Communicable Diseases. COVID-19 sentinel hospital surveillance update, week 51. 2021 Dec 28 [cited 2022 Jan 5]. https://www.nicd.ac.za/wp-content/uploads/2021/12/NICD-COVID-19-Weekly-Sentinel-Hospital-Surveillnace-update-Week-51-2021.pdf

6. South African Medical Research Council. Report on weekly deaths in South Africa, week 50. 2021 Dec 22 [cited 2022 Jan 5]. https://www.samrc.ac.za/reports/report-weekly-deaths-south-africa

7. Statistics South Africa. Statistical release P0302. Mid-year population estimates 2021. 2021 [cited 2022 Jan 5]. http://www.statssa.gov.za/?page_id=1854&PPN=P0302

8. National Institute for Communicable Diseases. COVID-19 weekly epidemiology brief week 3. 2022 Jan 26 [cited 2022 Jan 31]. https://www.nicd.ac.za/wp-content/uploads/2022/01/COVID-19-Weekly-Epidemiology-Brief-week-3-2022.pdf

9. Kleynhans J, Tempia S, Wolter N, von Gottberg A, Bhiman JN, Buys A, et al.; PHIRST-C Group. SARS-CoV-2 seroprevalence in a rural and urban household cohort during first and second waves of infections, South Africa, July 2020–March 2021. Emerg Infect Dis. 2021;27:3020–9. PubMed https://doi.org/10.3201/eid2712.211465

Appendix Table 1. Individuals seropositive for SARS-CoV-2 antibodies with adjusted seroprevalence and 95% credible intervals by blood draw, site and age, July 2020 – November 2021, South Africa*

Site	Age group, y	Seropositive/Tested (Seroprevalence*, 95% CrI)								
		BD1	BD2	BD3	BD4	BD5	BD6	BD7	BD8	BD9
Rural	<5	0/25 (4, 0–14)	1/49 (4, 0–14)	2/78 (4, 1–9)	14/87 (17, 10–25)	15/89 (18, 10–26)	14/88 (17, 10–25)	26/90 (29, 20–39)	33/77 (43, 32–54)	36/84 (43, 33–54)
	5–12	2/148 (2, 0–5)	5/169 (3, 0–5)	7/195 (4, 2–7)	29/202 (15, 10–20)	39/201 (20, 15–25)	42/202 (21, 16–27)	70/202 (35, 28–42)	120/200 (60, 53–67)	122/198 (62, 55–68)
	13–18	1/69 (3, 0–7)	5/72 (8, 0–7)	8/80 (11, 5–18)	20/82 (25, 16–35)	24/78 (31, 22–41)	24/75 (32, 23–43)	40/77 (52, 41–63)	59/73 (80, 71–88)	57/71 (80, 70–88)
	19–34	0/87 (1, 0–4)	5/88 (7, 0–4)	13/91 (15, 8–23)	33/91 (37, 27–47)	35/95 (37, 28–47)	34/89 (39, 29–49)	40/87 (46, 36–57)	48/81 (59, 48–70)	47/80 (59, 48–69)
	35–59	1/76 (2, 0–7)	5/77 (8, 0–7)	7/80 (10, 4–17)	23/81 (29, 20–39)	27/82 (33, 24–44)	27/84 (33, 23–43)	35/82 (43, 33–54)	49/80 (61, 50–72)	49/80 (61, 50–71)
	≥60	1/40 (5, 0–12)	4/39 (12, 0–12)	4/44 (11, 4–21)	8/45 (19, 9–32)	10/42 (25, 14–39)	10/41 (26, 14–39)	14/40 (36, 22–51)	16/37 (44, 28–60)	18/37 (49, 33–65)
	All	5/445 (1, 0–2)	25/494 (5, 0–2)	41/568 (7, 5–9)	127/588 (22, 18–25)	150/587 (26, 22–29)	151/579 (26, 23–30)	225/578 (39, 35–43)	325/548 (59, 55–64)	329/550 (60, 56–64)
Urban	<5	2/45 (6, 1–15)	5/50 (11, 4–21)	7/50 (15, 7–26)	8/44 (20, 9–32)	13/48 (28, 16–41)	14/45 (32, 19–46)	19/46 (42, 28–56)	25/45 (56, 41–70)	24/45 (53, 39–67)
	5–12	11/116 (10, 5–16)	17/116 (15, 9–22)	24/125 (20, 13–27)	32/123 (26, 19–34)	38/122 (32, 24–40)	52/125 (42, 33–50)	60/124 (49, 40–57)	75/124 (61, 52–69)	77/123 (63, 54–71)
	13–18	14/75 (19, 11–29)	19/75 (26, 17–36)	30/81 (37, 28–48)	35/77 (46, 35–57)	41/78 (53, 42–64)	44/77 (57, 46–68)	47/74 (63, 52–74)	59/74 (79, 69–88)	60/72 (83, 73–90)
	19–34	10/102 (10, 5–17)	17/98 (18, 11–26)	23/100 (23, 16–32)	29/99 (30, 21–39)	34/98 (35, 26–45)	44/94 (47, 37–57)	50/96 (52, 42–62)	65/95 (68, 59–77)	62/88 (70, 61–79)
	35–59	33/110 (30, 22–39)	45/110 (41, 32–50)	52/117 (45, 36–54)	61/112 (55, 45–64)	65/111 (59, 49–67)	70/111 (63, 54–72)	70/107 (65, 56–74)	82/104 (79, 70–86)	82/104 (79, 70–86)
	≥60	3/57 (7, 2–14)	6/55 (12, 5–22)	7/56 (14, 6–23)	16/55 (30, 19–42)	18/53 (35, 23–48)	23/53 (44, 31–57)	32/52 (61, 48–74)	33/51 (64, 51–77)	30/47 (64, 50–76)
	All	73/505 (14, 12–18)	109/504 (22, 18–25)	143/529 (27, 23–31)	181/510 (36, 31–40)	209/510 (41, 37–45)	247/505 (49, 45–53)	278/499 (56, 51–60)	339/493 (69, 65–73)	335/479 (70, 66–74)

*Adjusted for assay sensitivity and specificity. Blood draw (BD) 1, July 20–September 17, 2020; BD2, September 21–October 10, 2020; BD3, November 23–December 12, 2020; BD4, January 25–February 20, 2021; and BD5, March 22–April 11, 2021; BD6, May 20 – June 9, 2021; BD7, July 19 – August 5, 2021; BD8, September 13 – 25, 2021; BD9, November 15 – 27, 2021.

Appendix Table 2. Number and percentage of PHIRST-C participants with known vaccination status (N) vaccinated for SARS-CoV-2 by BD9 (November 15 – 27, 2021) in the rural and urban community, South Africa*

	Rural, No. (%)				Urban, No. (%)			
Age group, y	N	Not vaccinated	Partially vaccinated	Fully vaccinated	N	Not vaccinated	Partially vaccinated	Fully vaccinated
<5	93	93 (100)	0 (0)	0 (0)	49	49 (100)	0 (0)	0 (0)
5–12	205	205 (100)	0 (0)	0 (0)	125	125 (100)	0 (0)	0 (0)
13–18	83	82 (99)	1 (1)	0 (0)	78	72 (92)	3 (4)	3 (4)
19–34	101	91 (90)	2 (2)	8 (8)	96	72 (75)	8 (8)	16 (17)
35–59	85	51 (60)	9 (11)	25 (29)	111	59 (53)	5 (5)	47 (42)
≥60	42	16 (38)	10 (24)	16 (38)	53	16 (30)	6 (11)	31 (58)
All	609	538 (88)	22 (4)	49 (8)	512	393 (77)	22 (4)	97 (19)

*Partially vaccinated: one dose of Pfizer-BioNTech; Fully vaccinated: one dose of Johnson & Johnson or two doses of Pfizer-BioNTech; at time of blood collection

Appendix Table 3. Number and percentage of PHIRST-C participants with either serologic evidence of previous infection (at any draw 20 July, 2020 - November 27, 2021) or fully vaccinated by BD9 (November 15 – 27, 2021) or both in the rural and urban community, South Africa*

	n/N (%)	
Age group, y	Rural	Urban
<5	45/95 (47)	29/55 (53)
5–12	130/208 (63)	82/132 (62)
13–18	62/85 (73)	68/82 (83)
19–34	59/107 (55)	79/119 (66)
35–59	66/90 (73)	106/122 (87)
≥60	27/46 (59)	47/58 (81)
All	389/631 (62)	411/568 (72)

*Fully vaccinated: one dose of Johnson & Johnson or two doses of Pfizer-BioNTech; at time of blood collection

Appendix Table 4. The PHIRST-C Group

Name	Affiliation
Amelia Buys	Centre for Respiratory Diseases and Meningitis, National Institute for Communicable Diseases of the National Health Laboratory Service, Johannesburg, South Africa.
Anne von Gottberg	Centre for Respiratory Diseases and Meningitis, National Institute for Communicable Diseases of the National Health Laboratory Service, Johannesburg, South Africa. School of Pathology, Faculty of Health Sciences, University of the Witwatersrand, Johannesburg, South Africa.
Cheryl Cohen	Centre for Respiratory Diseases and Meningitis, National Institute for Communicable Diseases of the National Health Laboratory Service, Johannesburg, South Africa. School of Public Health, Faculty of Health Sciences, University of the Witwatersrand, Johannesburg, South Africa.
F. Xavier Gómez-Olivé	MRC/Wits Rural Public Health and Health Transitions Research Unit (Agincourt), Faculty of Health Sciences, School of Public Health, University of the Witwatersrand, Johannesburg, South Africa.
Floidy Wafawanaka	MRC/Wits Rural Public Health and Health Transitions Research Unit (Agincourt), Faculty of Health Sciences, School of Public Health, University of the Witwatersrand, Johannesburg, South Africa.
Jackie Kleynhans	Centre for Respiratory Diseases and Meningitis, National Institute for Communicable Diseases of the National Health Laboratory Service, Johannesburg, South Africa. School of Public Health, Faculty of Health Sciences, University of the Witwatersrand, Johannesburg, South Africa.
Jacques D. du Toit	MRC/Wits Rural Public Health and Health Transitions Research Unit (Agincourt), Faculty of Health Sciences, School of Public Health, University of the Witwatersrand, Johannesburg, South Africa.
Jinal N. Bhiman	Centre for Respiratory Diseases and Meningitis, National Institute for Communicable Diseases of the National Health Laboratory Service, Johannesburg, South Africa. School of Pathology, Faculty of Health Sciences, University of the Witwatersrand, Johannesburg, South Africa.
Jocelyn Moyes	Centre for Respiratory Diseases and Meningitis, National Institute for Communicable Diseases of the National Health Laboratory Service, Johannesburg, South Africa. School of Public Health, Faculty of Health Sciences, University of the Witwatersrand, Johannesburg, South Africa.
Kathleen Kahn	MRC/Wits Rural Public Health and Health Transitions Research Unit (Agincourt), Faculty of Health Sciences, School of Public Health, University of the Witwatersrand, Johannesburg, South Africa.
Kgaugelo Patricia Kgasago	Perinatal HIV Research Unit (PHRU), University of the Witwatersrand, Johannesburg, South Africa.
Limakatso Lebina	Perinatal HIV Research Unit (PHRU), University of the Witwatersrand, Johannesburg, South Africa. Africa Health Research Institute, Durban, South Africa. Africa Health Research Institute, KwaZulu-Natal, South Africa.
Linda de Gouveia	Centre for Respiratory Diseases and Meningitis, National Institute for Communicable Diseases of the National Health Laboratory Service, Johannesburg, South Africa.

Name	Affiliation
Maimuna Carrim	Centre for Respiratory Diseases and Meningitis, National Institute for Communicable Diseases of the National Health Laboratory Service, Johannesburg, South Africa. School of Pathology, Faculty of Health Sciences, University of the Witwatersrand, Johannesburg, South Africa.
Meredith L. McMorrow	Influenza Division, Centers for Disease Control and Prevention, Atlanta, Georgia, United States of America. Influenza Program, Centers for Disease Control and Prevention, Pretoria, South Africa.
Mignon du Plessis	Centre for Respiratory Diseases and Meningitis, National Institute for Communicable Diseases of the National Health Laboratory Service, Johannesburg, South Africa. School of Pathology, Faculty of Health Sciences, University of the Witwatersrand, Johannesburg, South Africa.
Neil A. Martinson	Perinatal HIV Research Unit (PHRU), University of the Witwatersrand, Johannesburg, South Africa. Johns Hopkins University Center for TB Research, Baltimore, Maryland, United States of America.
Nicole Wolter	Centre for Respiratory Diseases and Meningitis, National Institute for Communicable Diseases of the National Health Laboratory Service, Johannesburg, South Africa. School of Pathology, Faculty of Health Sciences, University of the Witwatersrand, Johannesburg, South Africa.
Retshidisitswe Kotane	Centre for Respiratory Diseases and Meningitis, National Institute for Communicable Diseases of the National Health Laboratory Service, Johannesburg, South Africa. School of Pathology, Faculty of Health Sciences, University of the Witwatersrand, Johannesburg, South Africa.
Stefano Tempia	Influenza Division, Centers for Disease Control and Prevention, Atlanta, Georgia, United States of America. Influenza Program, Centers for Disease Control and Prevention, Pretoria, South Africa. School of Public Health, Faculty of Health Sciences, University of the Witwatersrand, Johannesburg, South Africa. MassGenics, Atlanta, Georgia, United States of America.
Stephen Tollman	MRC/Wits Rural Public Health and Health Transitions Research Unit (Agincourt), School of Public Health, Faculty of Health Science, University of the Witwatersrand.
Tumelo Moloantoa	Perinatal HIV Research Unit (PHRU), University of the Witwatersrand, Johannesburg, South Africa.

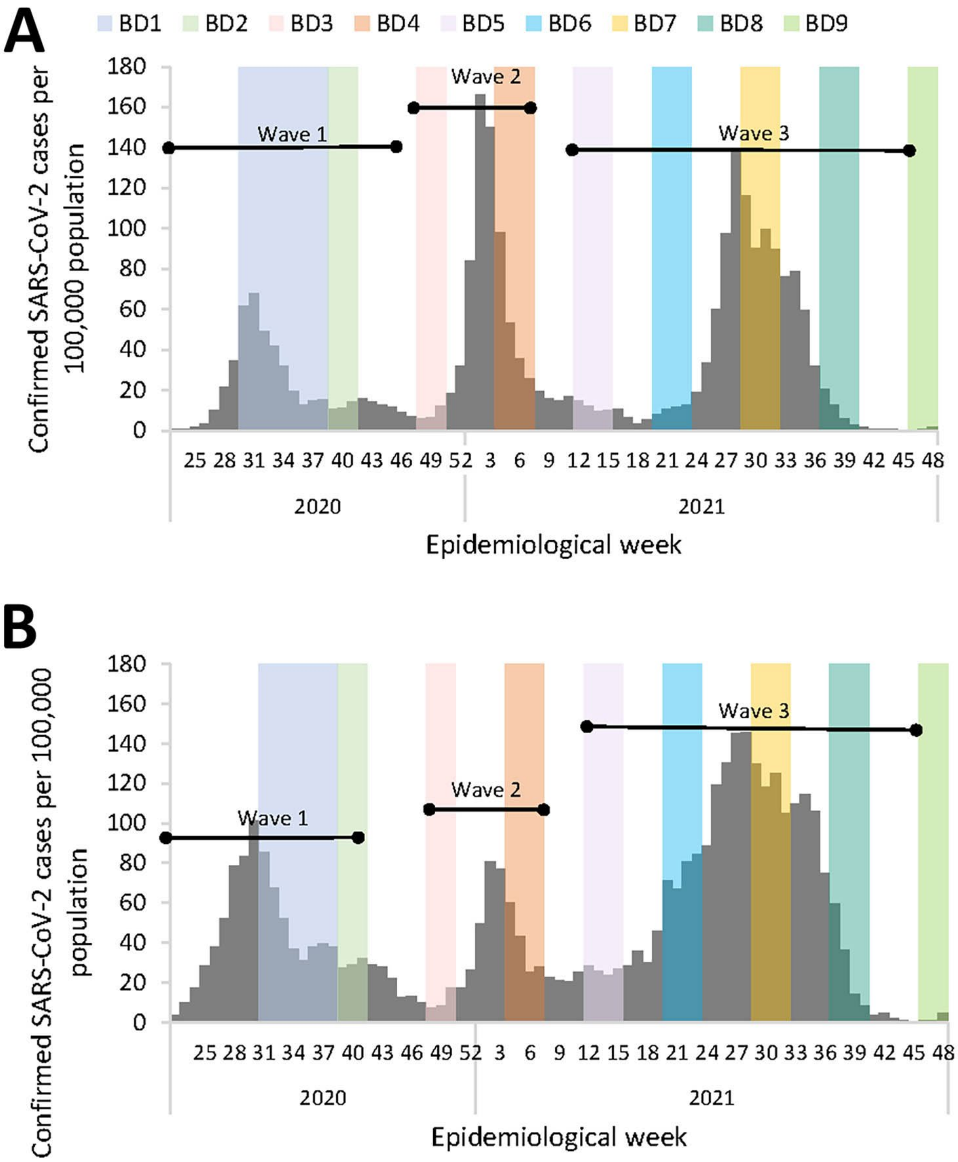

Appendix Figure 1. Timing of blood collection and district SARS-CoV-2 weekly incidence in a) rural community and b) urban community, March 2020 – November 2021, South Africa. November 23 – December 12, 2020; fourth draw (BD4) January, 25 – February 20, 2021; fifth draw (BD5) March 22 – April 11, 2021; sixth draw (BD6) May 20 – June 9, 2021; seventh draw (BD7) July 19 – August 5, 2021; eighth draw (BD8) September 13 – 25, 2021; ninth draw (BD9) November 15 – 27, 2021. Vertical lines represent 95% credible interval. Wave 1 (March 1 – November 21, 2020), 2 (November 22, 2020 – March 21, 2021) and 3 (March 22 – November 14, 2021) lines indicate period used for analysis. Laboratory-confirmed SARS-CoV-2 infections (reverse transcription polymerase chain reaction and antigen tests) as reported to the Notifiable Medical Conditions Surveillance System (NMCSS).

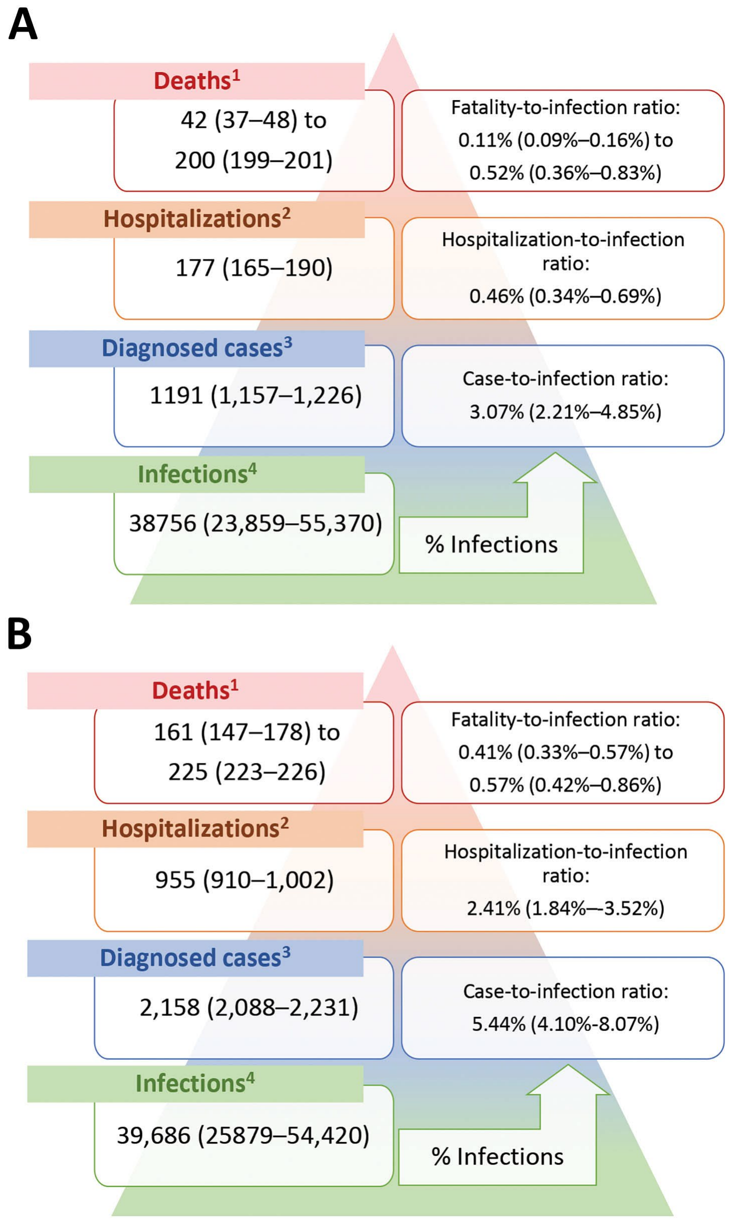

Appendix Figure 2. South African age-standardized SARS-CoV-2 infection, diagnosis, hospitalization and deaths per 100,000 population during wave 3 in the a) rural and b) urban community, March – November, 2021, South Africa. Standardized to South Africa mid-year population estimate for 2021. Wave 3: March 22 – November 14, 2021. [1]Minimum estimate: in-hospitalization deaths in districts based on COVID-19 Sentinel Hospital Surveillance report (DATCOV), maximum estimate: provincial excess deaths reported (South African Medical Research Council). [2]Hospitalizations in the district based on COVID-19 Sentinel Hospital Surveillance (DATCOV). [3]DiagnosedSARS-CoV-2 cases reported from districts from national SARS-CoV-2 surveillance (NMCSS). [1]Based on becoming seropositive or 2-fold increase in cutoff index (COI) between draw 5 and 9. [1]Values in bracket refer to 95% credible interval for infections and 95% confidence interval for all other estimates.

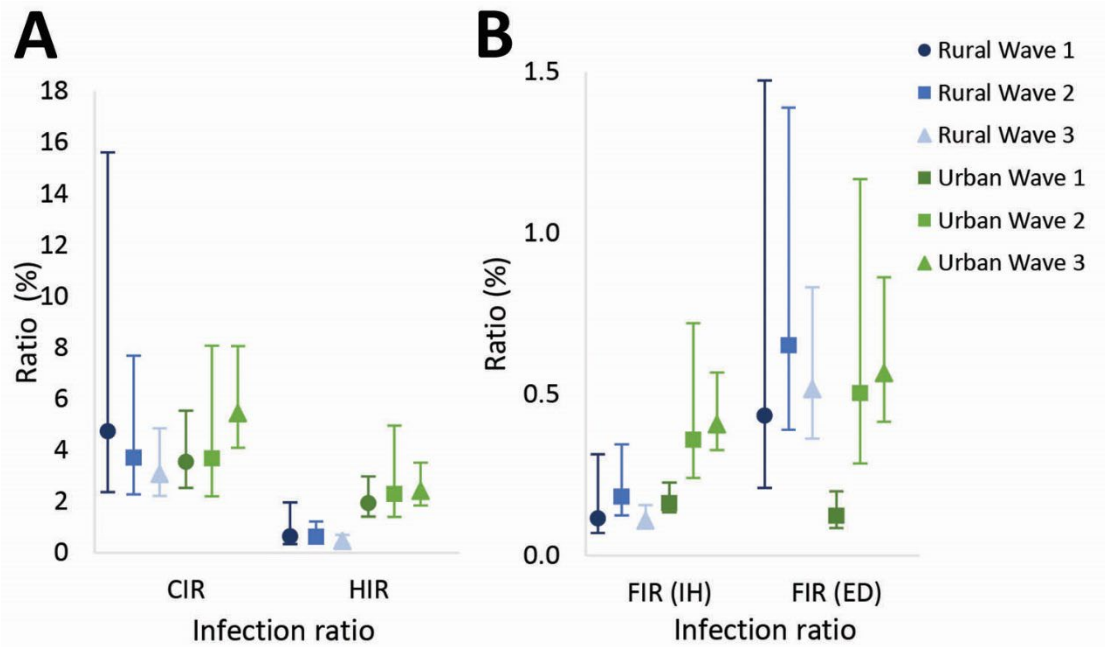

Appendix Figure 3. SARS-CoV-2 a) case-to-infection (CIR), hospitalization-to-infection (HIR) and b) in-hospital (IH) fatality-to-infection (FIR) and excess (ED) fatality-to-infection (FIR) ratios in a rural and urban community during the first, second and third wave of infections, March 2020 - November 2021, South Africa.
